# Paraquat Prohibition and Change in the Suicide Rate and Methods in South Korea

**DOI:** 10.1371/journal.pone.0128980

**Published:** 2015-06-02

**Authors:** Woojae Myung, Geung-Hee Lee, Hong-Hee Won, Maurizio Fava, David Mischoulon, Maren Nyer, Doh Kwan Kim, Jung-Yoon Heo, Hong Jin Jeon

**Affiliations:** 1 Department of Psychiatry, Depression Center, Samsung Medical Center, Sungkyunkwan University School of Medicine, Seoul, Korea; 2 Department of Information Statistics, Korea National Open University, Seoul, Korea; 3 Samsung Biomedical Research Institute, Samsung Medical Center, Seoul, Korea; 4 Depression Clinical and Research Program, Massachusetts General Hospital, Harvard Medical School, Boston, United States of America; 5 Department of Clinical Research Design and Evaluation and Department of Medical Device Management and Research, Samsung Advanced Institute for Health Sciences & Technology (SAIHST), Seoul, Korea; Medical University of Vienna, AUSTRIA

## Abstract

The annual suicide rate in South Korea is the highest among the developed countries. Paraquat is a highly lethal herbicide, commonly used in South Korea as a means for suicide. We have studied the effect of the 2011 paraquat prohibition on the national suicide rate and method of suicide in South Korea. We obtained the monthly suicide rate from 2005 to 2013 in South Korea. In our analyses, we adjusted for the effects of celebrity suicides, and economic, meteorological, and seasonal factors on suicide rate. We employed change point analysis to determine the effect of paraquat prohibition on suicide rate over time, and the results were verified by structural change analysis, an alternative statistical method. After the paraquat prohibition period in South Korea, there was a significant reduction in the total suicide rate and suicide rate by poisoning with herbicides or fungicides in all age groups and in both genders. The estimated suicide rates during this period decreased by 10.0% and 46.1% for total suicides and suicides by poisoning of herbicides or fungicides, respectively. In addition, method substitution effect of paraquat prohibition was found in suicide by poisoning by carbon monoxide, which did not exceed the reduction in the suicide rate of poisoning with herbicides or fungicides. In South Korea, paraquat prohibition led to a lower rate of suicide by paraquat poisoning, as well as a reduction in the overall suicide rate. Paraquat prohibition should be considered as a national suicide prevention strategy in developing and developed countries alongside careful observation for method substitution effects.

## Introduction

Suicide is a major global cause of death, representing the 13^th^ leading cause of death worldwide [1]. Establishing effective strategies for suicide prevention is an important public health priority. Several suicide prevention strategies have been implemented *i*.*e*. educational programs for the general public and professionals, screening of high-risk persons such as those with depression and alcohol use disorders, and a guideline for media reporting [2]. Reduction of access to lethal methods as a suicide prevention approach has been drawing attention [3]. The prohibition of toxic domestic gas [4], firearms [5], and suicide sites [3] are reportedly effective suicide prevention methods.

Paraquat (1,1-dimethyl-4-4-bipyridium dichloride, Gramoxone) is one of the most common herbicides causing suicide death. The paraquat poisoning is associated with a very high mortality rate, with 20 per million persons die from paraquat as a suicide method worldwide [6]. Risk mitigation measures—e.g., reducing the concentration and adding emetics—are reportedly inadequate. For this reason, the usage of paraquat has been banned in the European Union, Indonesia, and Kuwait.

According to the 2011 World Health Organization suicide prevention data, South Korea (Republic of Korea, ROK) is listed as having the 3^rd^ highest suicide rate among 105 countries. The lifetime prevalence of a suicide attempt in ROK was 3.2% from a nationwide community sample [7]. The annual suicide rate in ROK has gradually increased from 24.8 per 100,000 people in 2005 to 31.8 per 100,000 people in 2011. However, a substantial reduction in the suicide rate was shown in 2012 (28.1 per 100,000 people). The reasons for this decrease are not clear. One possible reason is the paraquat prohibition. In ROK, paraquat was the most frequent means of suicide in rural areas, accounting for 35.5% of all pesticide-related deaths [8]. It is easy to access in farmers’ stores, and is widely used year-round across the country due to its broad action as a herbicide. The government of ROK did not approve the license renewal of paraquat; hence the production of paraquat was banned November 23th, 2011. In addition, the distribution of paraquat to the market was prohibited subsequently in November 1^st^ 2012.

Given these factors, we assumed that the prohibition of paraquat would have had an impact on the overall suicide rate in ROK. On the other hand, we anticipated a method substitution effect [3, 9] in which other suicide methods would replace paraquat poisoning after the prohibition was implemented. If the method substitution effect sufficiently compensates for the decrease of suicide by paraquat poisoning, the total suicide rate should remain constant after prohibition. In this study, we hypothesized that the prohibition of paraquat would attenuate the rate of suicide by eliminating suicide deaths from poisoning with paraquat. Secondarily, we investigated whether the prohibition of paraquat would cause a decrease in total suicide rate and whether an alternative suicide method had emerged substitution after paraquat prohibition.

## Methods

### Suicide data

We obtained the rates of monthly completed suicides in the ROK from January 1 2005 to December 31 2013. Suicide data were derived from death records defined as suicides according to the International Classification of Diseases-10 (ICD-10) codes X60-X84, which include suicides from all causes. We obtained the suicide number from the data on the methods of suicide. Self-poisoning by toxic effect of herbicides or fungicides included suicide by poisoning with paraquat (ICD-10 code: T60.3). The data were thoroughly examined and verified by the Korea National Statistical Office (KNSO, http://kostat.go.kr/portal/english).

### Adjustment for celebrity suicides, and economic and meteorological data

We noted the periods following events of celebrity suicides in order to control for the influence. We defined celebrity suicide as a suicide reported for more than two weeks in news programs of the three major Korean national television networks [10]. Eleven suicides met the definition of celebrity suicide during the nine year period of this study. Additionally, we defined the affected period as 30 days after the first report of the celebrity suicide [10–11]. A monthly average of affected period days was used in the analysis. The economic data including economic sentiment index (ESI), unemployment rate, inflation rate and stock index valuations (KOSPI) were obtained from the KNSO. The meteorological data (sunlight hours and temperature) [12] were obtained from the Korea Meteorological Administration (KMA, http://web.kma.go.kr/eng).

### Ethics statement

Our research analyzes existing data that are publicly available in a manner that does not allow individual subjects to be identified; therefore ethics approval was deemed unnecessary.

### Statistical analysis

We adjusted seasonality of monthly suicide rate per 10 million people by X-13ARIMA-SEATS (http://www.census.gov/srd/www/x13as), a seasonal adjustment program developed by the US Census Bureau (S2 Text) [13]. All analyses were conducted by using the seasonal adjusted values. We firstly analyzed change of suicide rate by change point analysis [14]. Change point analysis is a widely utilized method used to detect multiple changes within a given time series. We used ‘cpt.mean’ function of ‘change point’ R package with the different penalty values according to the each suicide method due to their different incidence rates. Spearman correlation was used to investigate the association between suicide rate and candidate covariates. In addition, we applied structural change analysis of the suicide rate [15]. This alternative statistical method was employed to test the validity of the change point analysis, and to control for the covariates that were correlated with suicide rate. Ordinary least squares (OLS)-based cumulative sum (CUSUM) test and *F* statistics test were employed for distinguishing structural change. We estimated the breakpoints where Bayesian information criteria (BIC) were minimized [15]. We utilized a linear regression model with a dependent variable of natural logarithm-transformed monthly suicide rate per 10 million people. We checked variance inflation factors (VIF) in all regression models that were used in the structural change analysis (VIF < 5). All statistical analyses were performed with the R 3.1.0 public statistics software (http://www.r-project.org). Results were considered significant at a 2-tailed threshold of p < 0.05. The raw data (S1 Data) and the R package codes applicable to the analyses (S1 Text) were provided as additional information. The detailed explanation for structural change analysis is provided in S3 Text.

## Results

### Change point analyses

**Fig 1:**
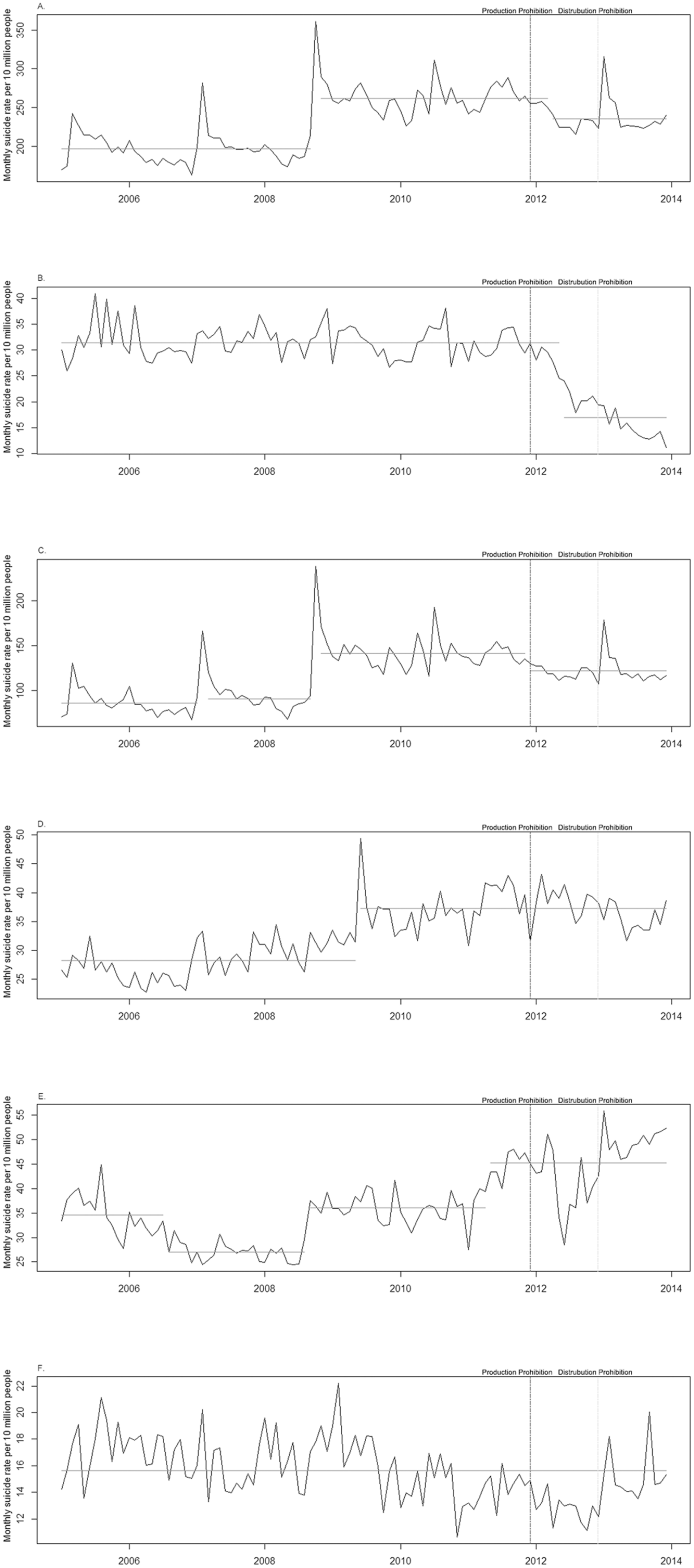
Change point analyses by year in South Korea. The Y-axis represents monthly suicide rate per 10 million people. The black line indicates the trend of seasonal adjusted suicide rate. The gray horizontal line indicates the estimated values of suicide rate by the change point analysis. Two vertical lines indicate the prohibition dates (boundary line for production prohibition and dashed line for distribution prohibition). (A) Total suicides. (B) Suicide by poisoning with herbicides or fungicides. (C) Suicide by hanging. (D) Suicide by falling/jumping. (E) Suicide by other poisoning. (F) Suicide by any other method.

Change point analysis for the total suicide rate demonstrated three change points: 1) September 2008, 2) October 2008, and 3) March 2012. September and October of 2008 were around the beginning of the 2008 financial crisis, and March 2012 was after the prohibition of paraquat production (Fig 1A). The total suicide rate decreased from 261·54 per 10 million people to 235.32 per 10 million people (10.0%, 26.23 per 10 million people) in March 2012, after the prohibition of paraquat production. Furthermore, change point analysis for the suicide rate of poisoning with herbicides or fungicides showed that suicide by this method decreased from 31.39 per 10 million people to 16.93 per 10 million people (46.1%, 14.46 per 10 million people, Fig 1B) in May 2012, after the prohibition of paraquat production. The suicide rate from poisoning with herbicides or fungicides continuously dropped after paraquat prohibition, a decline observed in all gender and age groups (S1 Fig).

**Fig 2:**
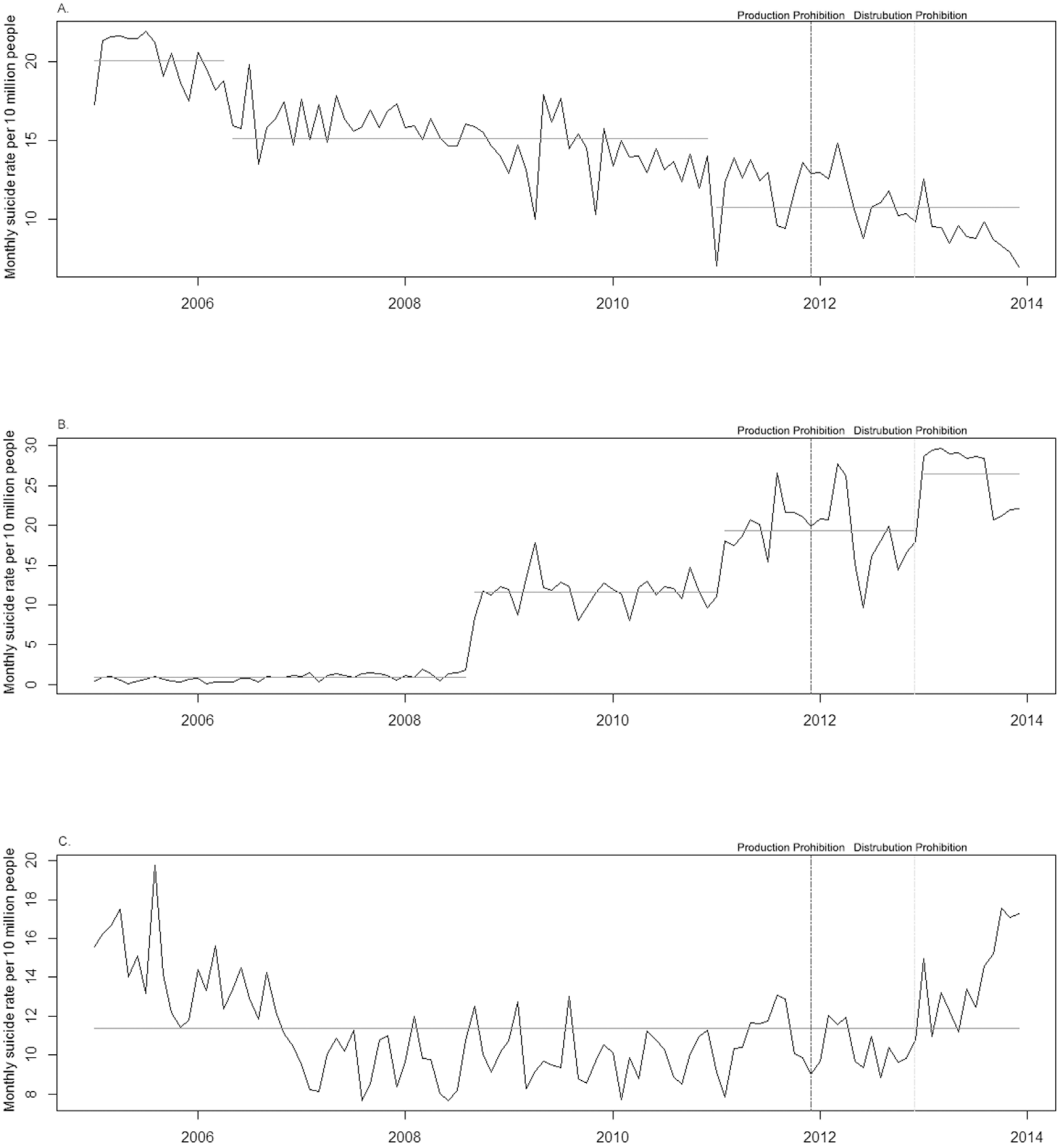
Change point analyses for method substitution effect by year in South Korea. (A) Suicide by poisoning with other agricultural chemicals. (B) Suicide by poisoning with carbon monoxide. (C) Suicide by poisoning with any other substance. The Y-axis represents monthly suicide rate per 10 million people. The black line indicates the trend of seasonal adjusted suicide rate. The gray horizontal line indicates the estimated values of suicide rate by the change point analysis. Two vertical lines indicate the prohibition dates (boundary line for production prohibition and dashed line for distribution prohibition).

Fig 1C showed that the rate of suicide by hanging decreased in November 2011, the month of prohibition of paraquat production (13.6% reduction, 19.21 per 10 million people). We did not find any change points for the rates of suicides by jumping/falling and any other methods of suicide around the paraquat prohibition dates (Fig 1D and 1F). However, the suicide rate from other poisoning abruptly increased around the time of prohibition of paraquat (Fig 1E): It increased by 24.7% (8.99 per 10 million people) in April 2011. This method substitution effect did not exceed the reduction in the suicide rate of poisoning with herbicides or fungicides (14.46 per 10 million people), so the reduction in the total suicide rate was sustained. Next, we conducted additional analyses to explore which specific substances among the other poisoning substituted suicide of poisoning with herbicides or fungicides. Fig 2A showed that the suicide rate of poisoning by other agricultural chemicals did not change around the paraquat prohibition dates. We found an abrupt increase of suicide rate of poisoning by carbon monoxide in December 2012, the initial month of prohibition of paraquat distribution (Fig 2B). Suicide rates of poisoning by any other substances were not changed during the study periods (Fig 2C).

### Structural change analyses

We tested whether the mean of the monthly suicide rate according to suicide methods changed over time by employing structural change analyses. These analyses have produced similar results with the change point analyses (S3A–S3F Fig). We found breakpoints for rates of total suicide (S3A Fig) and suicide by poisoning with herbicides or fungicides (S3B Fig) after paraquat production prohibition. The method substitution effect by other poisoning was also validated in structural change analyses (S3E Fig). We then repeated the structural change analysis with the covariates. Covariates significantly correlated with each suicide rates (S1 Table) were entered in structural change analyses. S3A–S3B Fig showed that reductions of total suicide and suicide by poisoning with herbicides or fungicides after paraquat prohibition were preserved after controlling for covariates, although the patterns of suicide rates were changed. In addition, the method substitution effect by other poisoning was maintained in this multivariate analysis (S3E Fig).

## Discussion

This was the first study to investigate the potential effect of the prohibition of paraquat on the whole national suicide rate in a developed countriy, ROK. This study presented three main findings. First, we found a marked decrease in suicide by poisoning with herbicides or fungicides in the population after the time of paraquat prohibition. Second, there was a prominent reduction in total suicide rate during this same period. Third, method substitution effect of paraquat prohibition was found in suicide by poisoning with carbon monoxide.

A previous study showed that the number of suicides in Samoa increased after the arrival of paraquat and was rapidly reduced after the control of paraquat [16]. Another study in Sri Lanka reported that the prohibition of toxic pesticides reduced the national suicide rate [17]. However, studies in developed countries are limited. A study conducted in France reported no change in the number of suicide attempts by paraquat poisoning before and after the European ban of paraquat [18]. However, this study was limited to data collected from one center, and did not examine nationwide data. Compared to previous studies, our results demonstrated that the effects of paraquat prohibition extended beyond developing countries to developed countries, by using nation-wide data.

Previous research has not investigated the reason for the reduction in the suicide rate of 2012 in ROK. In this study, we found evidence to support our hypothesis that the paraquat prohibition caused the 2012 reduction in ROK suicide rates with two lines of statistical evidence. First, the reduction of the total suicide rate occurred subsequent to paraquat prohibition. The time point of reduction of the total suicide rate was within four months of the date of paraquat prohibition. Second, the suicide rate reduction in poisoning with herbicides or fungicides following paraquat prohibition was remarkable in comparison to the total suicide rate. The change point analysis revealed a 46.1% decrease in the suicide rate of poisoning with herbicides or fungicides, while the total suicide rate decreased by 10.0%. These results were consistent with previous reports [4, 19–20] that national prohibition policies of highly lethal means commonly used in a country led to lower rates of suicide with those methods as well as overall suicide rates.

Paraquat has been known as among the most lethal agricultural chemical, with up to 70% mortality [21]. A retrospective study in ROK showed that only 38% of attempted suicide was by intentionally selected paraquat [6]. Unplanned attempters had precipitants for attempts such as familial conflict. It was also reported that methods such as the use of chemical agents or falling were three times more common in unplanned than planned attempters [22]. Moreover, van der Hoek et al. [23], reported that the intentional poisoning by paraquat is often the result of impulsive behavior rather than deliberate planning. A similar result was reported by a Sri Lankan study [24], where about 50% of self-poisoning patients ingested the poison less than 30 minutes after deciding to self-harm, and 85% of patients chose their poison based on availability. The benefit of mean prohibition depends on the lethality of the method. If a highly lethal method is not available, an individual with suicidal intention may not abandon their attempt completely, but may choose a less lethal method, so that their chance of survival increases [25]. Therefore, from a public health perspective, reducing access to lethal suicide methods can reduce overall suicide rates.

In our study, we analyzed the suicide rate related to different herbicides or fungicides instead of paraquat specific suicides. The fatalities of poisoning by agricultural chemicals varied in previous studies 6% to 75%, a rate which was relatively lower than paraquat alone (70%) [25]. There was a possibility after the policy change the attempters would replace paraquat with other less lethal agents due to unavailability, so that the number of complete suicides would decrease. Extended studies that include the number of suicide attempts will be helpful in verifying this replacement effect within the classes of agricultural chemical.

An interesting finding is that the marked reduction of suicide by poisoning with herbicides or fungicides started right after the prohibition of manufacturing, when paraquat was still sold in the market (Fig 1B). A stockpiling by the farmers could be a reason for this marked reduction after the prohibition of manufacturing. Paraquat was considered as most cost effective way to eradicate weeds among the farmers. After the government’s announcement of prohibition, the customers stockpiled paraquat, so it was in short supply within a month. This change of availability could affect the reduction of suicide by poisoning with herbicides or fungicides.

We found a method substitution effect by other suicide means. The rate of suicide by other poisoning rose around the time of paraquat prohibition. In the month of prohibition of paraquat distribution, moreover, the rate of suicide by carbon monoxide poisoning increased. In contrast, the rate of suicide by poisoning with other agricultural chemicals was constant. In line with previous studies,[26–27] the rate of suicide by carbon monoxide poisoning with charcoal briquettes increased significantly after the suicide of a famous actor (Ahn, Jae-hwan) found dead with burned charcoal briquettes in his vehicle on 8 September 2008 (Fig 2B). There is a general public perception that charcoal-burning suicide is painless and charcoal briquettes are easily accessible. There is a possibility that planned attempters intentionally select charcoal-burning suicide with awareness of the prohibition of lethal agricultural chemicals, so the increase of this method started after the suicide of the celebrity was more facilitated. Multilateral efforts such as limited access to charcoal, responsible media reporting, and detoxification of charcoal by modifying the raw material should be considered to reduce this method substitution effect.

An interesting finding was the decrease of suicide by hanging. One possible explanation was the extended effect of paraquat prohibition. The policy change might have aroused public opinion for suicide prevention, and improved the alertness among the high risk population. A national movement for suicide prevention should be considered as another explanation. The ROK government prepared and enacted a suicide prevention law in April 2013. This occurred after the reduction of 2012, but the increased awareness and preparation of the law could have contributed to the decreased suicide rate.

In addition, we should note that other factors besides paraquat prohibition may have contributed to the reduction of suicides in ROK. As previous research reported,[28–29] the suicide rate of ROK had been drastically dragged up due to global financial crisis. It was also represented in our result; Fig 1A showed an increased total suicide rate in September 2008 which marked the beginning of global financial crisis. There is a possibility that decrease in suicide by poisoning with herbicides or fungicides and reduction in total suicide rate could have been affected by economic recovery.[30].

This study has the following strengths. First, we employed rigorous seasonal adjustment by X-13ARIMA-SEATS [31]. Seasonal adjustment is a basic concept for analysis social and economical phenomena. The importance of precise seasonal adjustment, however has been commonly overlooked in the investigation of suicide [32]. Seasonal adjustment has a role in simplifying the data to be more easily interpreted without loss of information or spurious correlation [13]. Second, we confirmed the validity of the change point analysis by an alternative statistical method (structural change analysis). We therefore controlled for important covariates like celebrity suicide, economic factors and meteorological factors. A main limitation was the observational design. The results could be attributed to other unknown factors for which we did not assess. Another limitation is our relatively short timeframe. Although, our statistical approach enabled us to use monthly data instead of annually compiled data that would require a long time period to evaluate the effect of policy change [32], we could not investigate delayed method substitution effects that can be occur with longer time intervals. Another limitation is that paraquat specific data could not be analyzed due to the data classification of KNSO.

In conclusion, our findings expand the evidence for suicide prevention by lethal means prohibition. The ROK prohibition of paraquat led to reduction in the overall suicide rate, in addition to the rate of suicides by poisoning by agricultural chemicals. Our results support to paraquat prohibition as a national suicide prevention strategy, not only for developing countries but also for developed countries. On the other hand, we found a method substitution effect with carbon monoxide poisoning. This method substitution effect highlights the importance of continuous efforts to reduce the national suicide rate in ROK. Our statistical methodology can be helpful to additionally evaluate the effect of public health policy in a short-time period. Further longer-term studies are required to replicate these findings in developed countries.

## Supporting Information

S1 Data (CSV)

S1 Fig Change point analyses of suicide with poisoning of herbicides or fungicides according to gender and age groups. The Y-axis is monthly suicide rate per 10 million people. The black line indicates the trend of seasonal adjusted suicide rate. The red horizontal line indicates the estimated values of suicide rate by the change point analysis. Two vertical lines indicate the prohibition dates (production prohibition and distribution prohibition). (TIFF) (A) Male. (B) Female. (C) Age < 40. (D) Age: 40–59. (E) Age < 60.
(TIF)

S2 Fig Structural change analyses. The Y-axis is natural logarithm-transformed monthly suicide rate per 10 million people. The black line indicates the trend of seasonal adjusted suicide rate. The red dot line indicates the estimated values of suicide rate by the model with breakpoint(s). (TIFF) (A) Total suicides. (B) Suicide by poisoning with herbicides or fungicides. (C) Suicide by hanging. (D) Suicide by falling/jumping. (E) Suicide by other poisoning. (F) Suicide by any other method.
(TIF)

S3 Fig Structural change analyses with covariates. The Y-axis is natural logarithm-transformed monthly suicide rate per 10 million people. The black line indicates the trend of seasonal adjusted suicide rate. The red dot line indicates the estimated values of suicide rate by the model with breakpoint(s). (A) Total suicides. (B) Suicide by poisoning with herbicides or fungicides. (C) Suicide by hanging. (D) Suicide by falling/jumping. (E) Suicide by other poisoning. (F) Suicide by any other method.
(TIF)

S1 Table Correlation between suicide rate and candidate covariates. (DOCX)

S1 Text R package codes. (DOCX)

S2 Text Seasonal adjustment. (DOCX)

S3 Text The detailed explanation for structural change analysis. (DOCX)
