# Supplementary figures and images for: Paraquat Prohibition and Change in the Suicide Rate and Methods in South Korea
Source: PLoS One. 2015 Jun 2;10(6):e0128980. doi: 10.1371/journal.pone.0128980 (PMC4452788; doi:10.1371/journal.pone.0128980)

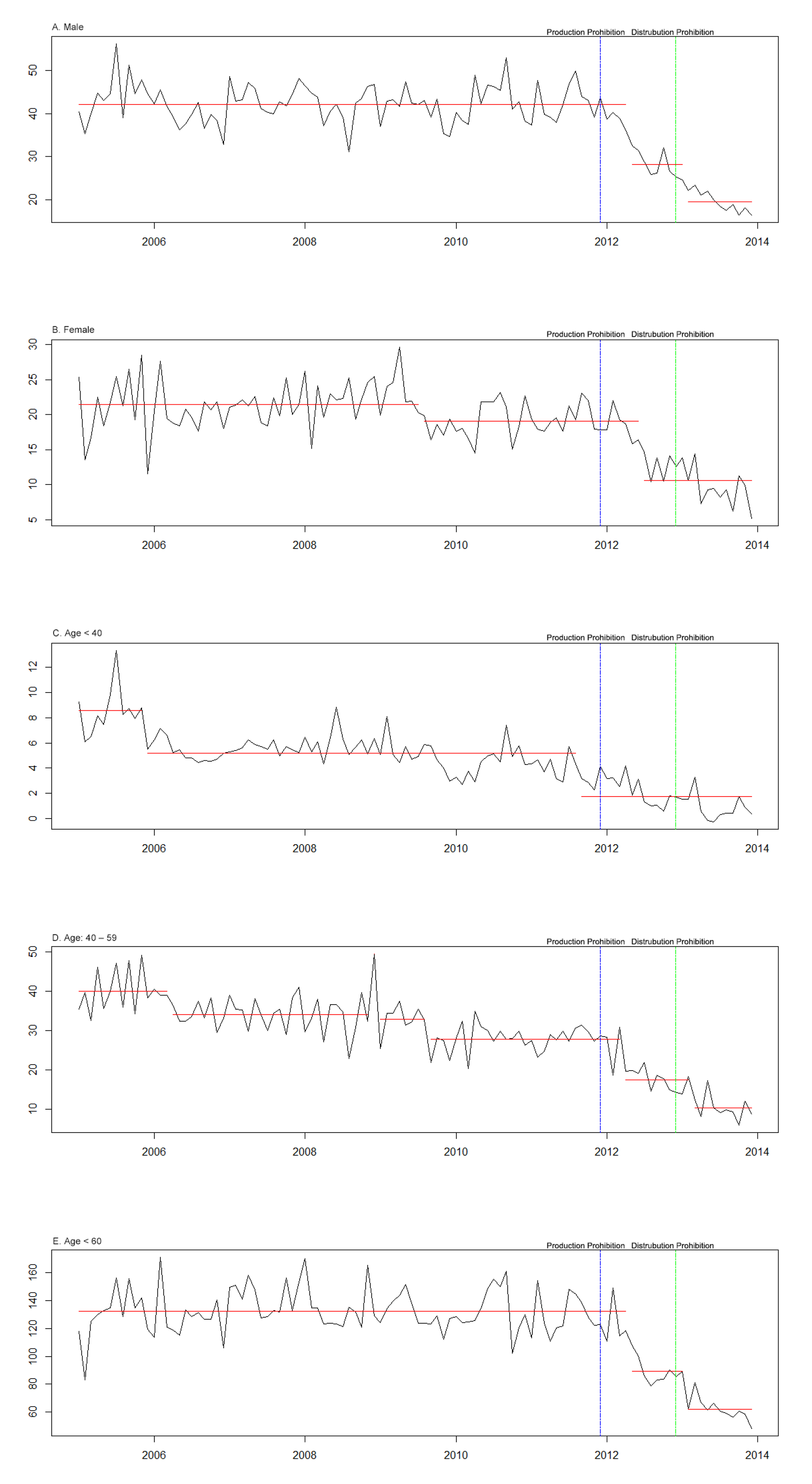

Supplement: S1 Fig — The Y-axis is monthly suicide rate per 10 million people. The black line indicates the trend of seasonal adjusted suicide rate. The red horizontal line indicates the estimated values of suicide rate by the change point analysis. Two vertical lines indicate the prohibition dates (production prohibition and distribution prohibition). (TIFF) (A) Male. (B) Female. (C) Age < 40. (D) Age: 40–59. (E) Age < 60. (TIF) [file pone.0128980.s002.tif]

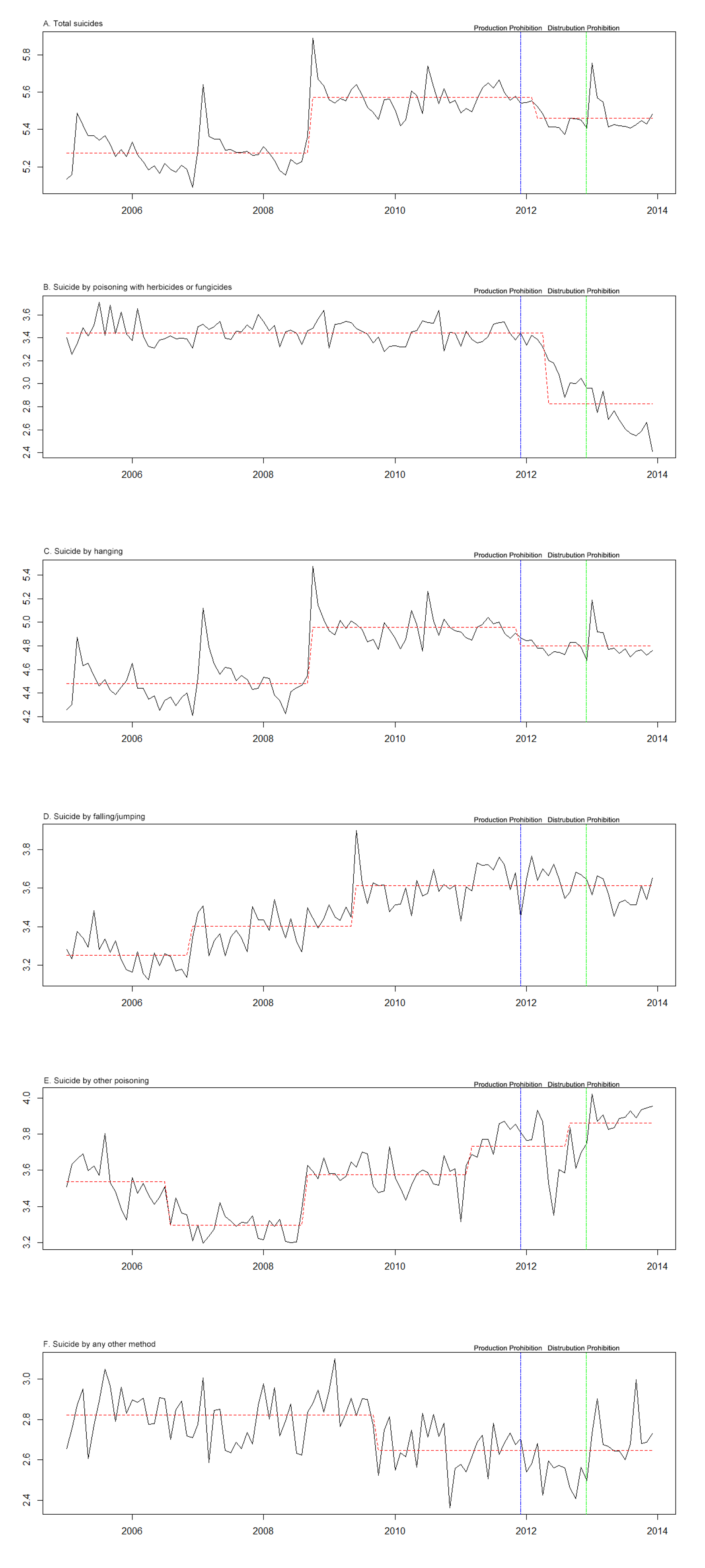

Supplement: S2 Fig — The Y-axis is natural logarithm-transformed monthly suicide rate per 10 million people. The black line indicates the trend of seasonal adjusted suicide rate. The red dot line indicates the estimated values of suicide rate by the model with breakpoint(s). (TIFF) (A) Total suicides. (B) Suicide by poisoning with herbicides or fungicides. (C) Suicide by hanging. (D) Suicide by falling/jumping. (E) Suicide by other poisoning. (F) Suicide by any other method. (TIF) [file pone.0128980.s003.tif]

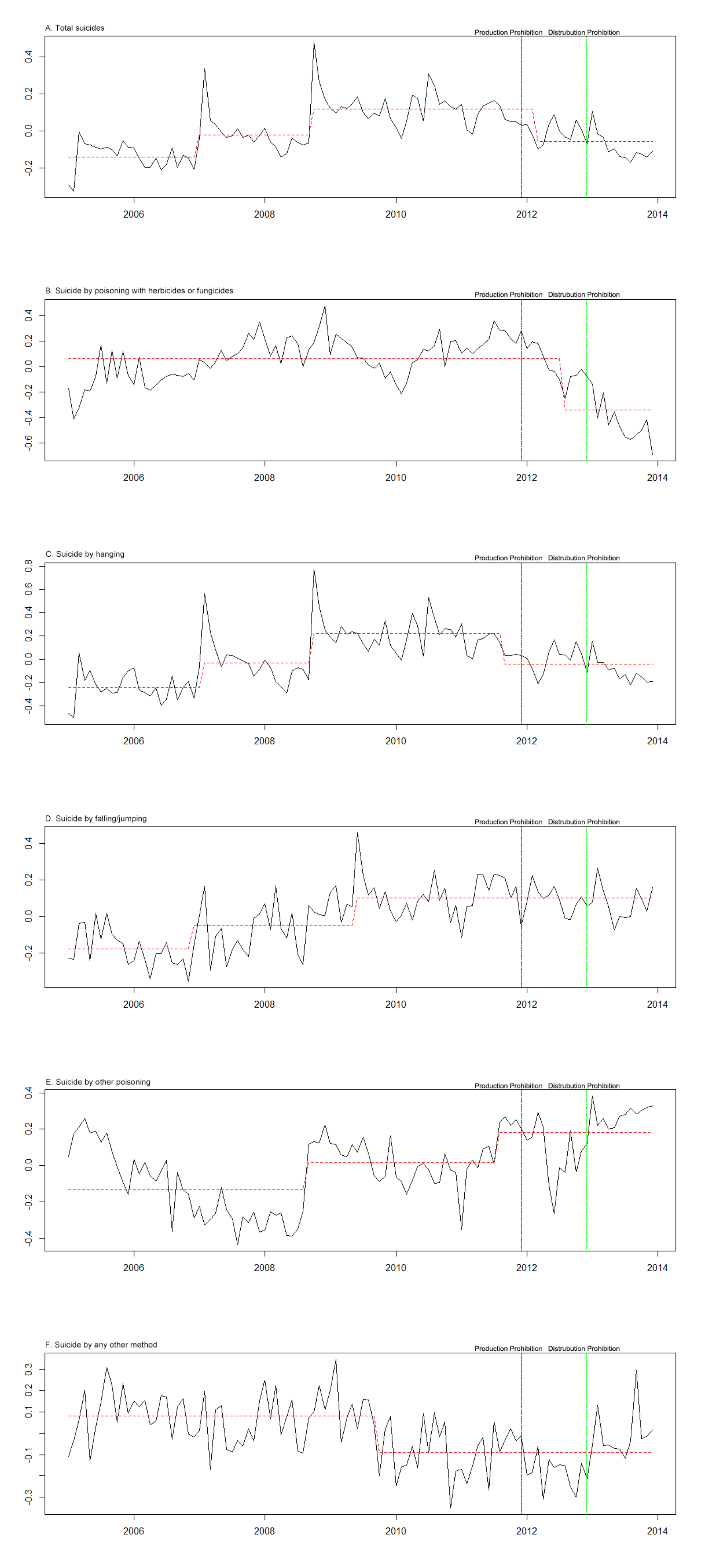

Supplement: S3 Fig — The Y-axis is natural logarithm-transformed monthly suicide rate per 10 million people. The black line indicates the trend of seasonal adjusted suicide rate. The red dot line indicates the estimated values of suicide rate by the model with breakpoint(s). (A) Total suicides. (B) Suicide by poisoning with herbicides or fungicides. (C) Suicide by hanging. (D) Suicide by falling/jumping. (E) Suicide by other poisoning. (F) Suicide by any other method. (TIF) [file pone.0128980.s004.tif]
