# Supplementary material for: Paraquat Prohibition and Change in the Suicide Rate and Methods in South Korea
Source: PLoS One. 2015 Jun 2;10(6):e0128980. doi: 10.1371/journal.pone.0128980 (PMC4452788; doi:10.1371/journal.pone.0128980)
Supplement: S1 Table — (DOCX) [file pone.0128980.s005.docx]

***Table S1:* Correlation between Suicide rate and Candidate Covariates**^*^

|  | Total Suicide | Suicide by poisoning with herbicides or fungicides | Suicide by Hanging | Suicide by Falling/Jumping | Other Poisoning | Suicide with Any Other Method |
| --- | --- | --- | --- | --- | --- | --- |
| Celebrity suicide |  |  |  |  |  |  |
| *rho* | 0•36 | 0•16 | 0•39 | 0•07 | 0•05 | 0•15 |
| *P* | <0•0001 | 0•09 | <0•0001 | 0•45 | 0•62 | 0•13 |
| ESI |  |  |  |  |  |  |
| *rho* | -0•17 | 0•27 | -0•13 | -0•21 | -0•45 | -0•01 |
| *P* | 0•07 | <0•01 | 0•19 | 0•03 | <0•0001 | 0•94 |
| Unemployment rate |  |  |  |  |  |  |
| *rho* | 0•10 | 0•23 | 0•11 | -0•15 | -0•08 | 0•26 |
| *P* | 0•30 | 0•02 | 0•25 | 0•13 | 0•41 | <0•01 |
| Inflation rate |  |  |  |  |  |  |
| *rho* | 0•16 | 0•49 | 0•16 | -0•01 | -0•17 | 0•07 |
| *P* | 0•10 | <0•0001 | 0•10 | 0•89 | 0•09 | 0•50 |
| Stock Index |  |  |  |  |  |  |
| *rho* | 0•29 | -0•40 | 0•29 | 0•63 | 0•37 | -0•56 |
| *P* | <0•01 | <0•0001 | <0•01 | <0•0001 | <0•0001 | <0•0001 |
| Sunlight hours |  |  |  |  |  |  |
| *rho* | -0•06 | 0•10 | -0•10 | 0•04 | -0•05 | 0•00 |
| *P* | 0•52 | 0•32 | 0•31 | 0•72 | 0•62 | 0•97 |
| Temperature |  |  |  |  |  |  |
| *rho* | -0•01 | -0•30 | -0•01 | 0•13 | 0•20 | -0•13 |
| *P* | 0•93 | <0•01 | 0•91 | 0•18 | 0•04 | 0•18 |

Abbreviation: ESI, economic sentiment index.

^*^ Spearman correlation was used.
