# Supplementary material for: Paraquat Prohibition and Change in the Suicide Rate and Methods in South Korea
Source: PLoS One. 2015 Jun 2;10(6):e0128980. doi: 10.1371/journal.pone.0128980 (PMC4452788; doi:10.1371/journal.pone.0128980)
Supplement: S1 Text — (DOCX) [file pone.0128980.s006.docx]

**Paraquat Prohibition and Change in the Suicide Rate and Methods in South Korea**

**Woojae Myung^1,2,3¶^, Geung-Hee Lee^2¶^, Hong-Hee Won^3^, Maurizio Fava^4^,
David Mischoulon^4^, Maren Nyer^4^, Doh Kwan Kim^1^, Jung-Yoon Heo^1^,
Hong Jin Jeon^1,4,5*^**

**1** Department of Psychiatry, Depression Center, Samsung Medical Center, Sungkyunkwan University School of Medicine, Seoul, Korea, **2** Department of Information Statistics, Korea National Open University, Seoul, Korea, **3** Samsung Biomedical Research Institute, Samsung Medical Center, Seoul, Korea, **4** Depression Clinical and Research Program, Massachusetts General Hospital, Harvard Medical School, Boston, USA, **5** Department of Clinical Research Design and Evaluation, and Department of Medical Device Management and Research, Samsung Advanced Institute for Health Sciences & Technology (SAIHST), Seoul, Korea

^*^Corresponding author

Email: jeonhj@skku.edu (HJJ)

^¶^These authors contributed equally to this work.

**Supplementary materials**

This material supplements but does not replace the content of the peer-reviewed paper published.

**Text S1: R package codes**

We have provided the R package codes with the corresponding raw data. These will enable readers to run their own independent analyses of our data.

**## Seasonal adjustment and Defining of variables**

# Open data

sdat = read.csv("Data S1.csv", sep = ",", header=TRUE)

sdat[,1] = as.Date(sdat[,1])

sdat = ts(sdat, start=c(2005,1), frequency=12)

celebrity = sdat[,12]

esi = sdat[,13]

unemployment = sdat[,14]

inflation = sdat[,15]

kospi = sdat[,16]

temp = sdat[,17]

sunshine = sdat[,18]

pesticides = sdat[,19]

# Month plot for checking seasonality (**Figure S1**)

monthplot(sdat[,2], ylab="Monthly suicide rate per 10 million people", labels = month.abb)

# Seasonal adjustment (https://www.census.gov/srd/www/x13as/x13down_pc.html)

Sys.setenv(X13_PATH = "c:/work/x13as")

require("seasonal")

mm_2= seas(sdat[,2], x11.appendfcst = "yes",x11 = list())

mm_3= seas(sdat[,3], x11.appendfcst = "yes",x11 = list())

mm_4= seas(sdat[,4], x11.appendfcst = "yes",x11 = list())

mm_5= seas(sdat[,5], x11.appendfcst = "yes",x11 = list())

mm_6= seas(sdat[,6], x11.appendfcst = "yes",x11 = list())

mm_7= seas(sdat[,7], x11.appendfcst = "yes",x11 = list())

mm_28= seas(sdat[,28], x11.appendfcst = "yes",x11 = list())

mm_29= seas(sdat[,29], x11.appendfcst = "yes",x11 = list())

mm_30= seas(sdat[,30], x11.appendfcst = "yes",x11 = list())

mm_55 = seas(sdat[,37], x11.appendfcst = "yes",x11 = list())

mm_56 = seas(sdat[,38], x11.appendfcst = "yes",x11 = list())

mm_57 = seas(sdat[,39], x11.appendfcst = "yes",x11 = list())

mm_58 = seas(sdat[,40], x11.appendfcst = "yes",x11 = list())

mm_59 = seas(sdat[,41], x11.appendfcst = "yes",x11 = list())

sa_total = final(mm_2)

sa_t603 = final(mm_3)

sa_hanging = final(mm_4)

sa_falling = final(mm_5)

sa_otherpoison = final(mm_6)

sa_other = final(mm_7)

sa_agri = final(mm_28)

sa_t58 = final(mm_29)

sa_etc = final(mm_30)

sa_t603_male = final( mm_55 )

sa_t603_female = final( mm_56 )

sa_t603_age1 = final( mm_57 )

sa_t603_age2 = final( mm_58 )

sa_t603_age3 = final( mm_59 )

sa_total = sdat[,8]

sa_t603 = sdat[,9]

sa_hanging = sdat[,10]

sa_falling = sdat[,11]

sa_otherpoison = sdat[,12]

sa_other = sdat[,13]

sa_agri = sdat[,34]

sa_t58 = sdat[,35]

sa_etc = sdat[,36]

# Seasonal Adjusted vs Unadjusted (**Figure S2)**

plot(mm_2, xlab="", ylab="Monthly suicide rate per 10 million people")

# Changing in suicide rate according to suicide method before and after paraquat restriction

plot(cbind(sa_total, sa_t603, sa_hanging), xlab="", main="")

abline(v=2011+11/12, lty=2, col=2)

abline(v=2012+10/11, lty=2, col=3)

plot(cbind(sa_falling, sa_otherpoison, sa_other), xlab="", main="")

abline(v=2011+11/12, lty=2, col=2)

abline(v=2012+10/11, lty=2, col=3)

**## Change point analysis**

# Total suicide (**Figure 1A**)

library(changepoint)

total.pelt = cpt.mean(sa_total, penalty="Manual", pen.value="1000*log(n) ",method="PELT")

plot(total.pelt, ylab="suicide", xlab="", main="Total")

sdat[cpts(total.pelt),1]

abline(v=2011+11/12, lty=6, col="blue")

abline(v=2012+10/11, lty=6, col="green")

# T60.3 suicide (**Figure 1B**)

t603.pelt = cpt.mean(sa_t603, penalty="Manual", pen.value="100*log(n)",method="PELT")

plot(t603.pelt, ylab="suicide", xlab="", main="T603")

sdat[cpts(t603.pelt),1]

abline(v=2011+11/12, lty=6, col="blue")

abline(v=2012+10/11, lty=6, col="green")

# Hanging suicide (**Figure 1C**)

hanging.pelt = cpt.mean(sa_hanging, penalty="Manual", pen.value="500*log(n)",method="PELT")

plot(hanging.pelt, ylab="suicide", xlab="", main="Hanging")

sdat[cpts(hanging.pelt),1]

abline(v=2011+11/12, lty=6, col="blue")

abline(v=2012+10/11, lty=6, col="green")

# Falling suicide (**Figure 1D**)

falling.pelt = cpt.mean(sa_falling, penalty="Manual", pen.value="100*log(n)",method="PELT")

plot(falling.pelt, ylab="suicide", xlab="", main="FALLING")

sdat[cpts(falling.pelt),1]

abline(v=2011+11/12, lty=6, col="blue")

abline(v=2012+10/11, lty=6, col="green")

# Other poisoning suicide (**Figure 1E**)

otherpoison.pelt = cpt.mean(sa_otherpoison, penalty="Manual", pen.value="100*log(n)",method="PELT")

plot(otherpoison.pelt, ylab="suicide", xlab="", main="OTHERPOISON")

sdat[cpts(otherpoison.pelt),1]

abline(v=2011+11/12, lty=6, col="blue")

abline(v=2012+10/11, lty=6, col="green")

# Other suicide (**Figure 1F**)

other.pelt = cpt.mean(sa_other, penalty="Manual", pen.value="100*log(n)",method="PELT")

plot(other.pelt, ylab="suicide", xlab="", main="OTHER")

sdat[cpts(other.pelt),1]

abline(v=2011+11/12, lty=6, col="blue")

abline(v=2012+10/11, lty=6, col="green")

# Other agricultural chemical suicide (**Figure 2A**)

agri.pelt = cpt.mean(sa_agri, penalty="Manual", pen.value="50*log(n)",method="PELT")

plot(agri.pelt, ylab="suicide", xlab="", main="Agricultural chemical")

sdat[cpts(agri.pelt),1]

abline(v=2011+11/12, lty=6, col="blue")

abline(v=2012+10/11, lty=6, col="green")

# T58 suicide (**Figure 2B**)

t58.pelt = cpt.mean(sa_t58, penalty="Manual", pen.value="50*log(n)",method="PELT")

plot(t58.pelt, ylab="suicide", xlab="", main="t58")

sdat[cpts(t58.pelt),1]

abline(v=2011+11/12, lty=6, col="blue")

abline(v=2012+10/11, lty=6, col="green")

# Poisoning suicide other than agricultural chemical and T58 (**Figure 2C**)

etc.pelt = cpt.mean(sa_etc, penalty="Manual", pen.value="50*log(n)",method="PELT")

plot(etc.pelt, ylab="suicide", xlab="", main=" Poisoning suicide other than agricultural chemical and T58")

sdat[cpts(etc.pelt),1]

abline(v=2011+11/12, lty=6, col="blue")

abline(v=2012+10/11, lty=6, col="green")

# T60.3 gender and age (**Figure S1**)

# Male (**Figure S1A**)

sa_t603_male.pelt=cpt.mean(sa_t603_male, penalty="Manual", pen.value="50*log(n) ",method="PELT")

plot(sa_t603_male.pelt, ylab="suicide", xlab="", main="sa_t603_male")

sdat[cpts(sa_t603_male.pelt),1]

abline(v=2011+11/12, lty=6, col="blue")

abline(v=2012+10/11, lty=6, col="green")

# Female (**Figure S1B**)

sa_t603_female.pelt = cpt.mean(sa_t603_female, penalty="Manual", pen.value="25*log(n) ",method="PELT")

plot(sa_t603_female.pelt, ylab="suicide", xlab="", main="sa_t603_female")

sdat[cpts(sa_t603_female.pelt),1]

abline(v=2011+11/12, lty=6, col="blue")

abline(v=2012+10/11, lty=6, col="green")

# Age: 0-39 (**Figure S1C**)

sa_t603_age1.pelt = cpt.mean(sa_t603_age1, penalty="Manual", pen.value="12.5*log(n) ",method="PELT")

plot(sa_t603_age1.pelt, ylab="suicide", xlab="", main="sa_t603_age1")

sdat[cpts(sa_t603_age1.pelt),1]

abline(v=2011+11/12, lty=6, col="blue")

abline(v=2012+10/11, lty=6, col="green")

# Age: 40-60 (**Figure S1D**)

sa_t603_age2.pelt = cpt.mean(sa_t603_age2, penalty="Manual", pen.value="25*log(n) ",method="PELT")

plot(sa_t603_age2.pelt, ylab="suicide", xlab="", main="sa_t603_age2")

sdat[cpts(sa_t603_age2.pelt),1]

abline(v=2011+11/12, lty=6, col="blue")

abline(v=2012+10/11, lty=6, col="green")

# Age: 60- (**Figure S1E**)

sa_t603_age3.pelt = cpt.mean(sa_t603_age3, penalty="Manual", pen.value="500*log(n) ",method="PELT")

plot(sa_t603_age3.pelt, ylab="suicide", xlab="", main="sa_t603_age3")

sdat[cpts(sa_t603_age3.pelt),1]

abline(v=2011+11/12, lty=6, col="blue")

abline(v=2012+10/11, lty=6, col="green")

**## Structure change analysis**

# CUSUM test

library(strucchange)

ocus.total <- efp(log(sa_total)~1, type="OLS-CUSUM")

plot(ocus.total, xlab="")

ocus.t603 <- efp(log(sa_t603)~1, type="OLS-CUSUM")

plot(ocus.t603, xlab="")

ocus.hanging <- efp(log(sa_hanging)~1, type="OLS-CUSUM")

plot(ocus.hanging, xlab="")

ocus.falling <- efp(log(sa_falling)~1, type="OLS-CUSUM")

plot(ocus.falling, xlab="")

ocus.otherpoison <- efp(log(sa_otherpoison)~1, type="OLS-CUSUM")

plot(ocus.otherpoison, xlab="")

ocus.other <- efp(log(sa_other)~1, type="OLS-CUSUM")

plot(ocus.other, xlab="")

# F statistics test

fs.total <- Fstats(log(sa_total) ~ 1)

plot(fs.total)

breakpoints(fs.total)

lines(breakpoints(fs.total))

fs.t603 <- Fstats(log(sa_t603) ~ 1)

plot(fs.t603)

breakpoints(fs.t603)

lines(breakpoints(fs.t603))

fs.hanging <- Fstats(log(sa_hanging) ~ 1)

plot(fs.hanging)

breakpoints(fs.hanging)

lines(breakpoints(fs.hanging))

fs.falling <- Fstats(log(sa_falling) ~ 1)

plot(fs.falling)

breakpoints(fs.falling)

lines(breakpoints(fs.falling))

fs.otherpoison <- Fstats(log(sa_otherpoison) ~ 1)

plot(fs.otherpoison)

breakpoints(fs.otherpoison)

lines(breakpoints(fs.otherpoison))

fs.other <- Fstats(log(sa_other) ~ 1)

plot(fs.other)

breakpoints(fs.other)

lines(breakpoints(fs.other))

# BIC and RSS: Decide the number of breakpoints and date

bp.total <- breakpoints(log(sa_total) ~ 1)

summary(bp.total)

plot(bp.total)

bp.t603 <- breakpoints(log(sa_t603) ~ 1)

summary(bp.t603)

plot(bp.t603)

bp.hanging <- breakpoints(log(sa_hanging) ~ 1)

summary(bp.hanging)

plot(bp.hanging)

bp.falling <- breakpoints(log(sa_falling) ~ 1)

summary(bp.falling)

plot(bp.falling)

bp.otherpoison <- breakpoints(log(sa_otherpoison) ~ 1)

summary(bp.otherpoison)

plot(bp.otherpoison)

bp.other <- breakpoints(log(sa_other) ~ 1)

summary(bp.other)

plot(bp.other)

# Apply structural change (**Figure S2**)

# Total suicide

bp1.total <- breakpoints(bp.total, breaks=2)

total.fac <- breakfactor(bp1.total)

fm.total <- lm(log(sa_total) ~ total.fac - 1)

plot(log(sa_total), xlab="")

lines(ts(predict(fm.total),start=c(2005,1), frequency=12), col=2, lty=2)

abline(v=2011+11/12, lty=6, col="blue")

abline(v=2012+10/11, lty=6, col="green")

# T60.3 suicide (**Figure S2B**)

bp1.t603 <- breakpoints(bp.t603, breaks=1)

t603.fac <- breakfactor(bp1.t603)

fm.t603 <- lm(log(sa_t603) ~ t603.fac - 1)

plot(log(sa_t603), xlab="")

lines(ts(predict(fm.t603),start=c(2005,1), frequency=12), col=2, lty=2)

abline(v=2011+11/12, lty=6, col="blue")

abline(v=2012+10/11, lty=6, col="green")

# Hanging suicide (**Figure S2C**)

bp1.hanging <- breakpoints(bp.hanging, breaks=2)

hanging.fac <- breakfactor(bp1.hanging)

fm.hanging <- lm(log(sa_hanging) ~ hanging.fac - 1)

plot(log(sa_hanging), xlab="")

lines(ts(predict(fm.hanging),start=c(2005,1), frequency=12), col=2, lty=2)

abline(v=2011+11/12, lty=6, col="blue")

abline(v=2012+10/11, lty=6, col="green")

# Falling suicide (**Figure S2D**)

bp1.falling <- breakpoints(bp.falling, breaks=2)

falling.fac <- breakfactor(bp1.falling)

fm.falling <- lm(log(sa_falling) ~ falling.fac - 1)

plot(log(sa_falling), xlab="")

lines(ts(predict(fm.falling),start=c(2005,1), frequency=12), col=2, lty=2)

abline(v=2011+11/12, lty=6, col="blue")

abline(v=2012+10/11, lty=6, col="green")

# Other poisoning suicide (**Figure S2E**)

bp1.otherpoison <- breakpoints(bp.otherpoison, breaks=4)

otherpoison.fac <- breakfactor(bp1.otherpoison)

fm.otherpoison <- lm(log(sa_otherpoison) ~ otherpoison.fac - 1)

plot(log(sa_otherpoison), xlab="")

lines(ts(predict(fm.otherpoison),start=c(2005,1), frequency=12), col=2, lty=2)

abline(v=2011+11/12, lty=6, col="blue")

abline(v=2012+10/11, lty=6, col="green")

# Other suicide (**Figure S2F**)

bp1.other <- breakpoints(bp.other, breaks=1)

other.fac <- breakfactor(bp1.other)

fm.other <- lm(log(sa_other) ~ other.fac - 1)

plot(log(sa_other), xlab="")

lines(ts(predict(fm.other),start=c(2005,1), frequency=12), col=2, lty=2)

abline(v=2011+11/12, lty=6, col="blue")

abline(v=2012+10/11, lty=6, col="green")

# CUSUM test after applying structural change

ocus.total.after <- efp(log(sa_total)~total.fac, type="OLS-CUSUM")

plot(ocus.total.after, xlab="")

ocus.t603.after <- efp(log(sa_t603)~t603.fac, type="OLS-CUSUM")

plot(ocus.t603.after, xlab="")

ocus.hanging.after <- efp(log(sa_hanging)~hanging.fac, type="OLS-CUSUM")

plot(ocus.hanging.after, xlab="")

ocus.falling.after <- efp(log(sa_falling)~falling.fac, type="OLS-CUSUM")

plot(ocus.falling.after, xlab="")

ocus.otherpoison.after <- efp(log(sa_otherpoison)~otherpoison.fac, type="OLS-CUSUM")

plot(ocus.otherpoison.after, xlab="")

ocus.other.after <- efp(log(sa_other)~other.fac, type="OLS-CUSUM")

plot(ocus.other.after, xlab="")

# Analysis of structural change with covariates (**Figure S3**)

# Total suicide (**Figure S3A**)

ocus.total.cov <- efp(log(sa_total)~celebrity+kospi, type="OLS-CUSUM")

plot(ocus.total.cov, xlab="")

fs.total.cov <- Fstats(log(sa_total) ~ celebrity+kospi)

plot(fs.total.cov)

breakpoints(fs.total.cov)

lines(breakpoints(fs.total.cov))

bp.total <- breakpoints(log(sa_total) ~ celebrity+kospi)

summary(bp.total)

plot(bp.total)

bp1.total.cov <- breakpoints(bp.total, breaks=3)

total.fac.cov <- breakfactor(bp1.total.cov)

res.total.cov <- ts(residuals(lm(log(sa_total) ~ celebrity+kospi)),start=c(2005,1), frequency=12)

fm.total.cov <- lm(res.total.cov ~ total.fac.cov - 1)

plot(res.total.cov, xlab="")

lines(ts(predict(fm.total.cov),start=c(2005,1), frequency=12), col=2, lty=2)

abline(v=2011+11/12, lty=6, col="blue")

abline(v=2012+10/11, lty=6, col="green")

library(car)

vif(lm(log(sa_total) ~ celebrity+kospi))

# T60.3 suicide (**Figure S3B**)

ocus.t603.cov <- efp(log(sa_t603)~esi+unemployment+kospi+inflation+sunshine, type="OLS-CUSUM")

plot(ocus.t603.cov, xlab="")

fs.t603.cov <- Fstats(log(sa_t603) ~ esi+unemployment+kospi+inflation+sunshine)

plot(fs.t603.cov)

breakpoints(fs.t603.cov)

lines(breakpoints(fs.t603.cov))

bp.t603 <- breakpoints(log(sa_t603) ~ esi+unemployment+kospi+inflation+sunshine)

summary(bp.t603)

plot(bp.t603)

bp1.t603.cov <- breakpoints(bp.t603, breaks=1)

t603.fac.cov <- breakfactor(bp1.t603.cov)

res.t603.cov <- ts(residuals(lm(log(sa_t603) ~ kospi+inflation+sunshine+pesticides)),start=c(2005,1), frequency=12)

fm.t603.cov <- lm(res.t603.cov ~ t603.fac.cov - 1)

plot(res.t603.cov, xlab="")

lines(ts(predict(fm.t603.cov),start=c(2005,1), frequency=12), col=2, lty=2)

abline(v=2011+11/12, lty=6, col="blue")

abline(v=2012+10/11, lty=6, col="green")

vif(lm(log(sa_t603) ~ kospi+inflation+sunshine+pesticides))

# Hanging suicide (**Figure S3C**)

ocus.hanging.cov <- efp(log(sa_hanging)~celebrity+kospi, type="OLS-CUSUM")

plot(ocus.hanging.cov, xlab="")

fs.hanging.cov <- Fstats(log(sa_hanging) ~ celebrity+kospi)

plot(fs.hanging.cov)

breakpoints(fs.hanging.cov)

lines(breakpoints(fs.hanging.cov))

bp.hanging <- breakpoints(log(sa_hanging) ~ celebrity+kospi)

summary(bp.hanging)

plot(bp.hanging)

bp1.hanging.cov <- breakpoints(bp.hanging, breaks=3)

hanging.fac.cov <- breakfactor(bp1.hanging.cov)

res.hanging.cov <- ts(residuals(lm(log(sa_hanging) ~ celebrity+kospi)),start=c(2005,1), frequency=12)

fm.hanging.cov <- lm(res.hanging.cov ~ hanging.fac.cov - 1)

plot(res.hanging.cov, xlab="")

lines(ts(predict(fm.hanging.cov),start=c(2005,1), frequency=12), col=2, lty=2)

abline(v=2011+11/12, lty=6, col="blue")

abline(v=2012+10/11, lty=6, col="green")

vif(lm(log(sa_hanging) ~ celebrity+kospi))

# Falling suicide (**Figure S3D**)

ocus.falling.cov <- efp(log(sa_falling)~esi+kospi, type="OLS-CUSUM")

plot(ocus.falling.cov, xlab="")

fs.falling.cov <- Fstats(log(sa_falling) ~ esi+kospi)

plot(fs.falling.cov)

breakpoints(fs.falling.cov)

lines(breakpoints(fs.falling.cov))

bp.falling <- breakpoints(log(sa_falling) ~ esi+kospi)

summary(bp.falling)

plot(bp.falling)

bp1.falling.cov <- breakpoints(bp.falling, breaks=2)

falling.fac.cov <- breakfactor(bp1.falling.cov)

res.falling.cov <- ts(residuals(lm(log(sa_falling) ~ esi+kospi)),start=c(2005,1), frequency=12)

fm.falling.cov <- lm(res.falling.cov ~ falling.fac.cov - 1)

plot(res.falling.cov, xlab="")

lines(ts(predict(fm.falling.cov),start=c(2005,1), frequency=12), col=2, lty=2)

abline(v=2011+11/12, lty=6, col="blue")

abline(v=2012+10/11, lty=6, col="green")

vif(lm(log(sa_falling) ~ esi+kospi))

# Other poisoning suicide (**Figure S3E**)

ocus.otherpoison.cov <- efp(log(sa_otherpoison)~esi+kospi+sunshine, type="OLS-CUSUM")

plot(ocus.otherpoison.cov, xlab="")

fs.otherpoison.cov <- Fstats(log(sa_otherpoison) ~ esi+kospi+sunshine)

plot(fs.otherpoison.cov)

breakpoints(fs.otherpoison.cov)

lines(breakpoints(fs.otherpoison.cov))

bp.otherpoison <- breakpoints(log(sa_otherpoison) ~ esi+kospi+sunshine)

summary(bp.otherpoison)

plot(bp.otherpoison)

bp1.otherpoison.cov <- breakpoints(bp.otherpoison, breaks=2)

otherpoison.fac.cov <- breakfactor(bp1.otherpoison.cov)

res.otherpoison.cov <- ts(residuals(lm(log(sa_otherpoison) ~ esi+kospi+sunshine)),start=c(2005,1), frequency=12)

fm.otherpoison.cov <- lm(res.otherpoison.cov ~ otherpoison.fac.cov - 1)

plot(res.otherpoison.cov, xlab="")

lines(ts(predict(fm.otherpoison.cov),start=c(2005,1), frequency=12), col=2, lty=2)

abline(v=2011+11/12, lty=6, col="blue")

abline(v=2012+10/11, lty=6, col="green")

vif(lm(log(sa_otherpoison) ~ esi+kospi+sunshine))

# Other suicide (**Figure S3F**)

ocus.other.cov <- efp(log(sa_other)~unemployment+kospi, type="OLS-CUSUM")

plot(ocus.other.cov, xlab="")

fs.other.cov <- Fstats(log(sa_other) ~ unemployment+kospi)

plot(fs.other.cov)

breakpoints(fs.other.cov)

lines(breakpoints(fs.other.cov))

bp.other <- breakpoints(log(sa_other) ~ unemployment+kospi)

summary(bp.other)

plot(bp.other)

bp1.other.cov <- breakpoints(bp.other, breaks=1)

other.fac.cov <- breakfactor(bp1.other.cov)

res.other.cov <- ts(residuals(lm(log(sa_other) ~ unemployment+kospi)),start=c(2005,1), frequency=12)

fm.other.cov <- lm(res.other.cov ~ other.fac.cov - 1)

plot(res.other.cov, xlab="")

lines(ts(predict(fm.other.cov),start=c(2005,1), frequency=12), col=2, lty=2)

abline(v=2011+11/12, lty=6, col="blue")

abline(v=2012+10/11, lty=6, col="green")

vif((lm(log(sa_other) ~ unemployment+kospi))
