# Supplementary material for: Paraquat Prohibition and Change in the Suicide Rate and Methods in South Korea
Source: PLoS One. 2015 Jun 2;10(6):e0128980. doi: 10.1371/journal.pone.0128980 (PMC4452788; doi:10.1371/journal.pone.0128980)
Supplement: S2 Text — (DOCX) [file pone.0128980.s007.docx]

**Paraquat Prohibition and Change in the Suicide Rate and Methods in South Korea**

**Woojae Myung^1,2,3¶^, Geung-Hee Lee^2¶^, Hong-Hee Won^3^, Maurizio Fava^4^,
David Mischoulon^4^, Maren Nyer^4^, Doh Kwan Kim^1^, Jung-Yoon Heo^1^,
Hong Jin Jeon^1,4,5*^**

**1** Department of Psychiatry, Depression Center, Samsung Medical Center, Sungkyunkwan University School of Medicine, Seoul, Korea, **2** Department of Information Statistics, Korea National Open University, Seoul, Korea, **3** Samsung Biomedical Research Institute, Samsung Medical Center, Seoul, Korea, **4** Depression Clinical and Research Program, Massachusetts General Hospital, Harvard Medical School, Boston, USA, **5** Department of Clinical Research Design and Evaluation, and Department of Medical Device Management and Research, Samsung Advanced Institute for Health Sciences & Technology (SAIHST), Seoul, Korea

^*^Corresponding author

Email: jeonhj@skku.edu (HJJ)

^¶^These authors contributed equally to this work.

**Supplementary materials**

This material supplements but does not replace the content of the peer-reviewed paper published.

**Text S2: Seasonal adjustment**

Monthly suicide rate showed seasonal variation with peaks in the spring months and troughs in the fall months. The curves showed monthly suicide rate per 10 million people, and a line for the monthly average across each month’s data.


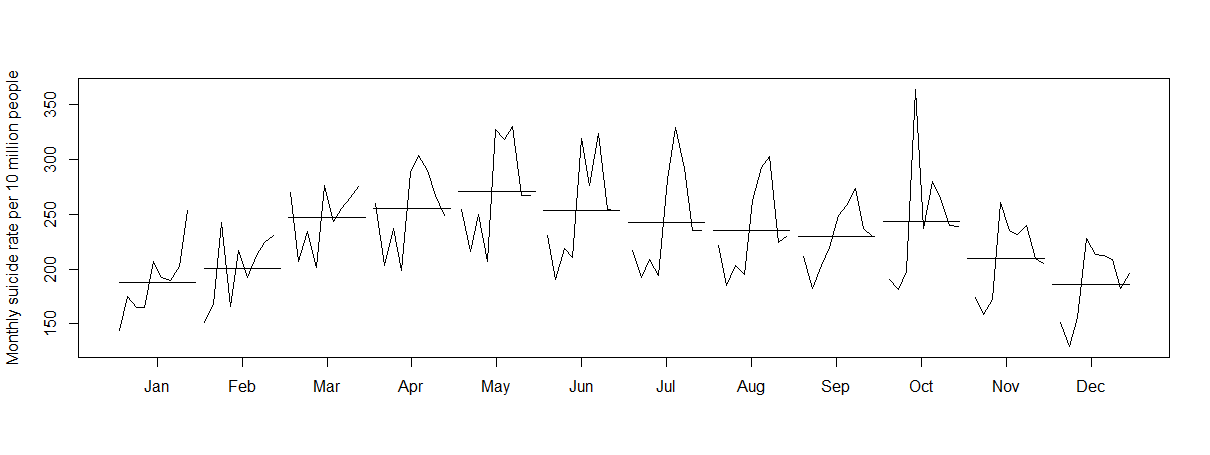


**- Monthly plot of suicide rate per 10 million people, through 2005-2012 –**

The seasonal adjusted suicide rate is shown in below figure. The seasonal adjusted suicide rate (red bold line) had more stable values with several spikes than unadjusted trend (black thin line), and the oscillations due to seasonal effect were smoothened out.


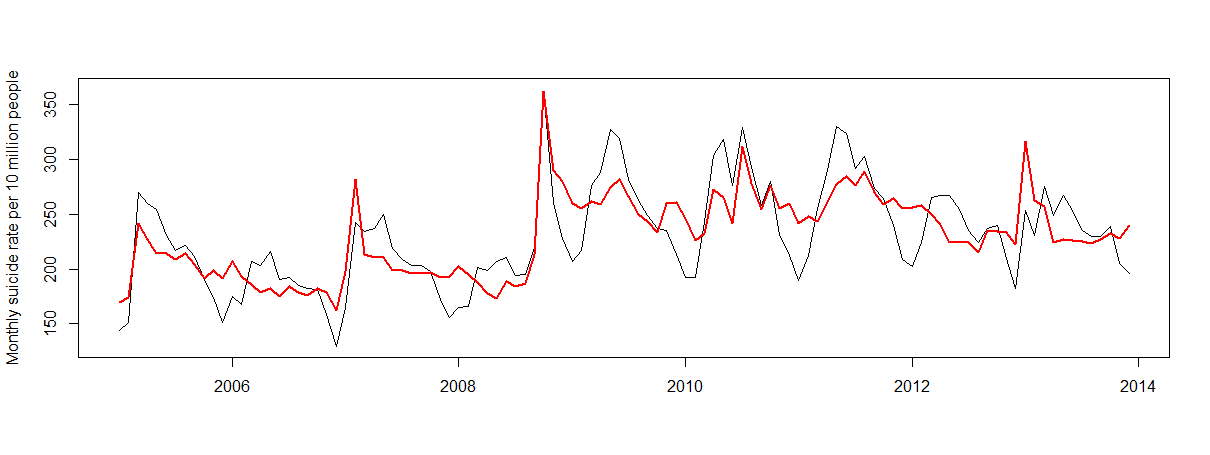


**- Trend of the monthly suicide rate per 10 million people, through 2005-2012 -**
