# Supplementary material for: Paraquat Prohibition and Change in the Suicide Rate and Methods in South Korea
Source: PLoS One. 2015 Jun 2;10(6):e0128980. doi: 10.1371/journal.pone.0128980 (PMC4452788; doi:10.1371/journal.pone.0128980)
Supplement: S3 Text — (DOCX) [file pone.0128980.s008.docx]

**Paraquat Prohibition and Change in the Suicide Rate and Methods in South Korea**

**Woojae Myung^1,2,3¶^, Geung-Hee Lee^2¶^, Hong-Hee Won^3^, Maurizio Fava^4^,
David Mischoulon^4^, Maren Nyer^4^, Doh Kwan Kim^1^, Jung-Yoon Heo^1^,
Hong Jin Jeon^1,4,5*^**

**1** Department of Psychiatry, Depression Center, Samsung Medical Center, Sungkyunkwan University School of Medicine, Seoul, Korea, **2** Department of Information Statistics, Korea National Open University, Seoul, Korea, **3** Samsung Biomedical Research Institute, Samsung Medical Center, Seoul, Korea, **4** Depression Clinical and Research Program, Massachusetts General Hospital, Harvard Medical School, Boston, USA, **5** Department of Clinical Research Design and Evaluation, and Department of Medical Device Management and Research, Samsung Advanced Institute for Health Sciences & Technology (SAIHST), Seoul, Korea

^*^Corresponding author

Email: jeonhj@skku.edu (HJJ)

^¶^These authors contributed equally to this work.

**Supplementary materials**

This material supplements but does not replace the content of the peer-reviewed paper published.

**Text S3: The detailed explanation for structural change analysis**

We tested whether the mean of the monthly total suicide rate changes over time. We consider the standard linear regression model with a dependent variable of natural logarithm-transformed monthly suicide rate per 10 million people.


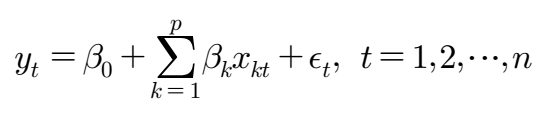


If we assume that there are *m* breakpoints, where the coefficients shift from on stable regression relationship to a different one. Thus, there are *m+1* segments in which the regression coefficients are constant, and the model can be rewritten as


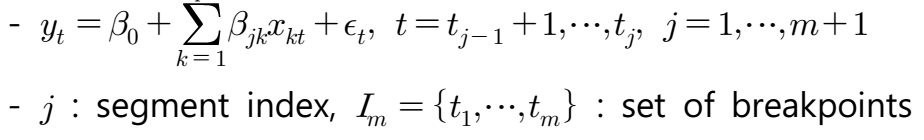


Then, we found m by using OLS-based CUSUM test and F statistics test, and $\beta_{\mathrm{jk}}$ was calculated by method of least squares.

The Ordinary least squares (OLS)-based cumulative sum (CUSUM) test showed a peak around 2008 which exceeded the boundaries of significance level (red lines) and indicated a structural shift. In other words, estimated monthly suicide rate by the linear regression model was not constant over the time.


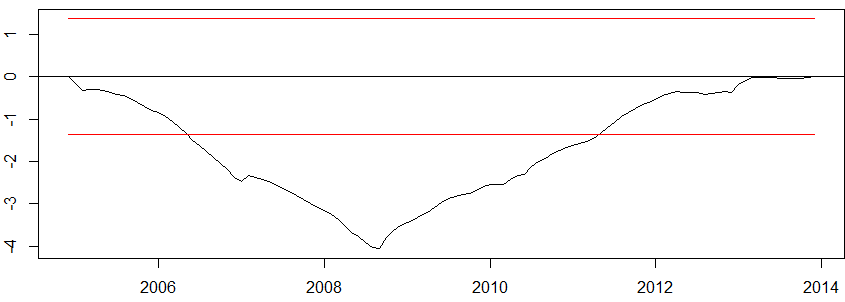


**- OLS based CUSUM test for total suicide -**

In addition, the *F* statistics showed similar results. The *F* statistics showed a high peak at around 2008. These results indicate more than one breakpoint can be obtained.


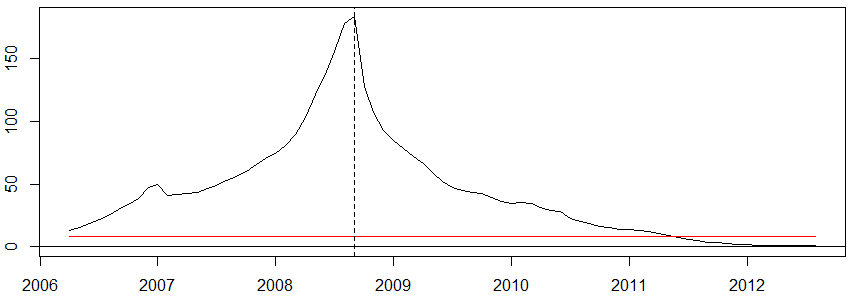


**- The F statistics test for total suicide -**

We compared multiple models with several breakpoints based on where Bayesian information criteria (BIC).


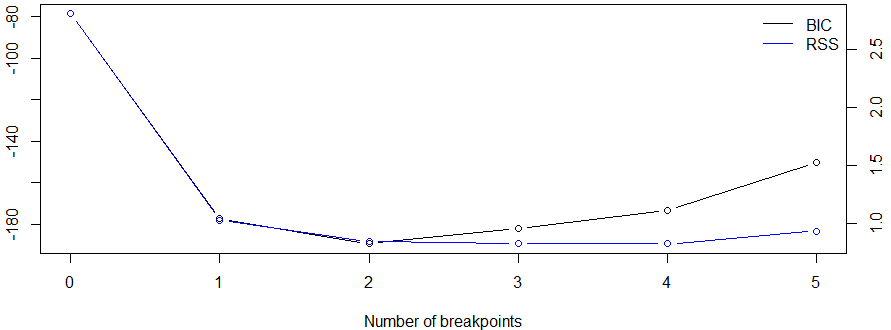


- **Bayesian information criteria (BIC) and residual sum of squares (RSS) for models according to the number of breakpoints -**

BIC for the model with two breakpoints showed the lowest value of BIC, therefore we decided in favor of a model with two breakpoints which were September 2008, and March 2012. The OLS-based CUSUM test with applied breakpoints confirmed the stability of the model.

The model with two breakpoints estimated that the total suicide rate was increased by 35·2%; it changed from 195·20 per 10 million people (exp[5·27]) to 263·03 per 10 million people (exp[5·57]) in September 2008 at the start of the 2008 financial crisis (**Figure S3A**). In addition, the model showed that total suicide rate decreased from 263·03 per 10 million people (exp[5·57]) to 234.49 per 10 million people (exp[5·46]) in March 2012 after the date of prohibition of paraquat production (28·54 per 10 million people, 10·9% decrease).

We then repeated the structural change analysis with the covariates that were correlated with suicide rate. Celebrity suicide and KOSPI were selected for covariates of the total suicide model by Spearman correlation. **Figure S4A** showed the result of multivariable analysis. Similar to the univariate analyses, the rate of total suicide was decreased after the prohibition of paraquat production. See the R package codes for our analyses in Text S1. More detail description of structure change analysis is available in paper of Zeileis A (Zeileis A, Kleiber C, Krämer W, Hornik K. Testing and dating of structural changes in practice. *Comput Stat Data Anal* 2003; **44:** 109-23.).
